# Diabetes and total knee arthroplasty: A nationwide analysis of complications, hospitalization outcomes and revision burden

**DOI:** 10.1002/ksa.12696

**Published:** 2025-05-12

**Authors:** Yaara Berkovich, Ela Cohen Nissan, David Maman, Michael Tobias Hirschmann, Yaniv Yonai, Yaniv Steinfeld, Yaron Berkovich

**Affiliations:** ^1^ Technion Israel Institute of Technology Haifa Israel; ^2^ Department of Orthopedics Carmel Medical Center Haifa Israel; ^3^ Department of Orthopaedic Surgery and Traumatology Kantonsspital Baselland (Bruderholz, Liestal, Laufen) Bruderholz Switzerland

**Keywords:** complications, diabetes, NIS, revision surgery, surgical outcomes, total knee arthroplasty

## Abstract

**Purpose:**

Total knee arthroplasty (TKA) is a frequently performed surgical procedure aimed at reducing pain, improving mobility, and restoring function in patients with advanced knee osteoarthritis. As patients undergoing TKA age, the prevalence of comorbidities, particularly diabetes, continues to rise. This study assesses post‐operative complications, healthcare costs and hospital length of stay (LOS) among diabetic patients undergoing primary and revision TKA using nationwide data from the NIS database, employing propensity score matching (PSM) to minimize confounding variables. We hypothesized that diabetic patients undergoing TKA would experience higher complication rates, greater healthcare costs and longer hospital stays compared to non‐diabetic controls.

**Methods:**

This retrospective cohort analysis utilized data from the Nationwide Inpatient Sample from 2016 to 2019, including a total of 2,602,484 TKA patients: 561,340 with type 2 diabetes and 2,041,144 without diabetes. PSM was applied to create balanced cohorts, adjusting for baseline demographic and clinical differences. Outcomes analyzed included LOS, total hospitalization charges, post‐operative complications and revision surgery rates. Statistical significance was set at *p* < 0.05.

**Results:**

Following PSM, diabetic patients exhibited significantly higher risks for post‐operative complications, including sepsis, heart failure and surgical site infections, compared to matched non‐diabetic controls. Diabetic patients also incurred significantly greater hospital charges ($64,694 vs. $59,952, *p* < 0.001). In revision TKA cases, diabetic patients demonstrated slightly longer LOS (3.5 days vs. 3.0 days, *p* < 0.001) and higher total hospital charges ($101,457 vs. $96,614, *p* = 0.015).

**Conclusions:**

Diabetic patients undergoing TKA experience significantly higher complication rates, hospital charges and revision surgery burden. Orthopaedic surgeons and perioperative teams should implement personalized perioperative management strategies, including optimized glycaemic control, cardiovascular risk assessment and infection prevention measures, to mitigate these risks and improve clinical outcomes.

**Levels of Evidence:**

Level III.

**Abbreviations**
AKI: acute kidney injury
HMO: Health Maintenance Organization
ICD‐10: International Classification of Diseases, 10th Revision
LOS: length of stay
NIS: Nationwide Inpatient Sample
OA: osteoarthritis
RR: risk ratio
TKA: total knee arthroplasty

## INTRODUCTION

As the age of patients undergoing total knee arthroplasty (TKA) increases, a significant number of these patients present with multiple comorbidities [8, 9, 12, 19, 23, 24]. One particularly relevant comorbidity is diabetes mellitus, the prevalence of which has increased dramatically in recent decades, from approximately 180 million cases in 1980 to over 422 million cases globally in 2014. Recent studies have shown that diabetes not only serves as a risk factor for developing osteoarthritis (OA) but also contributes to more severe disease presentations, potentially impacting surgical outcomes following TKA [10, 11, 17, 18, 25, 34, 39].

Currently, glycated haemoglobin A1c (HbA1c) serves as both a diagnostic tool and a method for monitoring diabetes management [9, 15, 33, 42]. Elevated HbA1c levels (>7.5%) are associated with increased healthcare costs and complications following TKA. Therefore, recent guidelines emphasize optimizing glycaemic control preoperatively to enhance patient outcomes and reduce complications [12, 16, 26, 30, 35, 37].

Previous studies exploring post‐operative outcomes following TKA in diabetic patients have mostly been limited to smaller, single‐centre studies, hindering definitive conclusions due to limited statistical power and generalizability [6, 11, 20, 22, 24, 40]. Leveraging large‐scale, nationwide data such as the Nationwide Inpatient Sample (NIS), which provides extensive inpatient records across the United States, can overcome these limitations by offering comprehensive and robust data for analysis [21].

This study aims to evaluate post‐operative complications, hospitalization outcomes, and the revision burden among diabetic patients undergoing TKA. We hypothesize that diabetic patients will exhibit higher complication rates, increased healthcare costs and longer hospital stays compared to non‐diabetic patients.

We hypothesized that diabetic patients undergoing TKA have increased risks of post‐operative complications, healthcare costs and longer hospital stays compared to non‐diabetic patients.

## MATERIALS AND METHODS

### Data set acquisition and study period

This retrospective cohort study utilized data from the NIS, the largest publicly available all‐payer inpatient care database in the United States. Data from 1 January 2016 to 31 December 2019 were analyzed, representing the most recent pre‐COVID‐19 pandemic period. The selection of these years was intentional, as data from 2020 onward was affected significantly by the COVID‐19 pandemic, altering hospital practices and substantially reducing the number of inpatient TKA procedures. By 2022, a large proportion of TKA surgeries shifted to outpatient or day‐surgery settings, limiting comparability. Thus, data from 2016 to 2019 represent the most recent and reliable data set for inpatient TKA analysis. While the NIS database is robust, its hospital‐based administrative data nature poses inherent limitations such as potential coding inaccuracies or misclassification. We minimized these risks through rigorous data selection and validation criteria.

### Patient identification and exclusions

Patients undergoing primary TKA were identified using specific International Classification of Diseases, 10th Revision (ICD‐10) procedure codes. Exclusion criteria for the primary TKA analysis included non‐elective admissions, patients younger than 18 years, and cases involving revision surgeries. Additionally, a separate sub‐analysis focusing specifically on revision TKA surgeries was conducted using respective ICD‐10 procedure codes.

### Statistical analyses and propensity score matching (PSM)

Statistical analyses were performed using SPSS version 26 (IBM Corp.) and MATLAB 2024 (MathWorks Inc.). Descriptive analyses included chi‐square tests for categorical variables and independent sample *t* tests for continuous variables. A significance threshold was set at *p* < 0.05.

To minimize potential confounding factors and selection bias, PSM was employed. Propensity scores were calculated using logistic regression, incorporating demographic variables (age, gender and payer type), hospital characteristics and comorbidities (e.g., hypertension, dyslipidemia, obstructive sleep apnoea, chronic kidney disease and congestive heart failure). Patients with type 2 diabetes were matched 1:1 to non‐diabetic patients using nearest‐neighbour matching without replacement, setting a calliper width of 0.01. Matching balance was rigorously assessed using standardized mean differences (SMDs), ensuring all matched covariates demonstrated an SMD < 0.1, indicating minimal residual imbalance between groups.

### Handling of missing data and sensitivity analysis

The NIS data set had minimal missing data (<2%) for variables included in the analysis. Missing data were addressed using listwise deletion. To ensure robustness and reliability, sensitivity analyses were conducted, confirming that outcomes were consistent across different scenarios for handling missing data.

### Comorbidity and outcome identification

Comorbidities were identified using ICD‐10 codes to assess the prevalence and distribution of medical conditions among patients with and without type 2 diabetes. Statistical analyses were conducted to evaluate the relationship between these comorbidities and key clinical outcomes. The outcomes examined included length of stay (LOS), total hospitalization costs and a comprehensive range of 18 different post‐operative complications, including infections, pulmonary embolism, acute kidney injury (AKI), pneumonia, cardiovascular events, sepsis, respiratory failure, wound dehiscence and ileus.

### Revision surgery analysis

Revision TKA cases were identified using specific ICD‐10 procedure codes. A total of 223,240 revision TKA surgeries were analyzed, comprising 167,950 patients without type 2 diabetes (75.2%) and 55,290 patients with type 2 diabetes (24.8%). Outcomes evaluated included revision aetiology (e.g., infection and mechanical loosening), total charges and LOS.

### Ethical aspects

This study was exempt from institutional review board approval due to the use of de‐identified data from the NIS. Informed consent was not required as the NIS database does not include patient‐identifiable information.

## RESULTS

**Figure 1:**
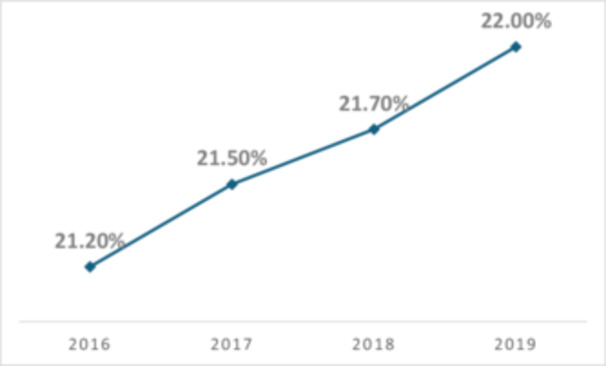
Annual proportion of type 2 diabetes patients among all total knee arthroplasty procedures (2016–2019).

There has been a notable trend in the increasing proportion of TKA patients with type 2 diabetes. As shown in Figure 1, the data indicate a gradual yet significant upward trend in the percentage of TKA patients with type 2 diabetes, rising from 21.20% in 2016 to 22.00% in 2019, with a *p* value of less than 0.001.

### Demographic, payer characteristics and comorbidity comparison between TKA patients with and without type 2 diabetes

As shown in Table 1, patients with type 2 diabetes accounted for 21.6% of all TKA procedures. These patients were slightly older, with an average age of 67.4 years compared to 66.6 years in those without diabetes (*p* < 0.001). The proportion of female patients was lower in the diabetic group (58.3% vs. 62.5%, *p* < 0.001). In terms of payer characteristics, Medicare was the primary expected payer for a larger proportion of diabetic patients (62% vs. 56%, *p* < 0.001), while private insurance was less common (29.5% vs. 36.2%).

**Table 1:** Demographic, payer characteristics and comorbidity comparison in TKA patients with and without type 2 diabetes.

Parameter	Patients without type 2 diabetes	Patients with type 2 diabetes	Significance
Total surgeries	2,041,144 (78.4%)	561,340 (21.6%)	–
Average age (years)	66.6	67.4	*p* < 0.001
Female (%)	62.5	58.3	*p* < 0.001
Primary expected payer—Medicare (%)	56	62	*p* < 0.001
Primary expected payer—Medicaid (%)	4.2	4.8	
Primary expected payer—private including HMO (%)	36.2	29.5	
Primary expected payer—self‐pay (%)	0.5	0.4	
Primary expected payer—no charge (%)	0	0.1	
Primary expected payer—other (%)	3.1	3.2	
Hypertension (%)	56.4	70.7	*p* < 0.001
Dyslipidemia (%)	41.7	64.4	*p* < 0.001
Obstructive sleep apnoea (%)	11.4	20.1	*p* < 0.001
Chronic anaemia (%)	5.6	6.9	*p* < 0.001
Alcohol abuse (%)	1	0.7	*p* < 0.001
Osteoporosis (%)	4.2	3.3	*p* < 0.001
Parkinson's disease (%)	0.6	0.6	*p* = 0.958
Alzheimer's disease (%)	0.2	0.2	*p* < 0.001
Chronic kidney disease (%)	5.2	13.5	*p* < 0.001
Congestive heart failure (%)	1	2.3	*p* < 0.001
Chronic lung disease (%)	5.4	8.4	*p* < 0.001
Rheumatoid arthritis (%)	3.5	3.3	*p* < 0.001

Abbreviations: HMO, Health Maintenance Organization; TKA, total knee arthroplasty.

Comorbidities were notably higher in the diabetic group, with significant differences in the prevalence of hypertension, dyslipidemia and obstructive sleep apnoea. Chronic kidney disease and congestive heart failure were also significantly more common among patients with diabetes (*p* < 0.001). Osteoporosis and rheumatoid arthritis were slightly less prevalent in this group, and Parkinson's disease showed no difference between the two cohorts.

### Propensity score‐matched analysis of comorbidities in TKA patients with and without type 2 diabetes

To address baseline differences and potential selection bias, a propensity score‐matched analysis was performed. This statistical method ensured that patients with and without type 2 diabetes undergoing TKA were matched based on key baseline characteristics. PSM creates statistically equivalent groups, balancing variables such as age, gender and primary payer type, thereby minimizing confounding factors and improving the reliability of comparisons.

Table 2 provides a detailed comparison of comorbidities between propensity score‐matched groups, each consisting of 561,313 patients. The results reveal no statistically significant differences in demographic and clinical parameters, including age, gender, payer distribution and the prevalence of most comorbidities. Conditions such as hypertension, dyslipidemia, chronic kidney disease and obstructive sleep apnoea were similar between the two groups, with *p* values indicating no significant differences.

**Table 2:** Propensity score‐matched comparison of demographics and comorbidities in TKA patients with and without type 2 diabetes.

Parameter	Patients without type 2 diabetes	Patients with type 2 diabetes	Significance
Total surgeries (%)	561,313	561,313	–
Average age (years)	67.5	67.4	*p* = 0.58
Female (%)	58.4	58.3	*p* = 0.93
Primary expected payer—Medicare (%)	62.3	62	*p* = 0.22
Primary expected payer—Medicaid (%)	4.6	4.8	
Primary expected payer—private including HMO (%)	29.5	29.5	
Primary expected payer—self‐pay (%)	0.4	0.4	
Primary expected payer—no charge (%)	0	0.1	
Primary expected payer—other (%)	3.2	3.2	
Hypertension (%)	71	70.7	*p* = 0.12
Dyslipidemia (%)	64.5	64.4	*p* = 0.35
Obstructive sleep apnoea (%)	19.8	20.1	*p* = 0.18
Chronic anaemia (%)	6.7	6.9	*p* = 0.28
Alcohol abuse (%)	0.7	0.7	*p* = 0.54
Osteoporosis (%)	3.5	3.3	*p* = 0.11
Parkinson's disease (%)	0.5	0.6	*p* = 0.07
Alzheimer's disease (%)	0.1	0.2	*p* = 0.05
Chronic kidney disease (%)	13.4	13.5	*p* = 0.20
Congestive heart failure (%)	2.3	2.3	*p* = 0.85
Chronic lung disease (%)	8.6	8.4	*p* = 0.14
Rheumatoid arthritis (%)	3.3	3.3	*p* = 0.64

Abbreviations: HMO, Health Maintenance Organization; TKA, total knee arthroplasty.

These findings highlight the effectiveness of the matching process in creating comparable groups, allowing for a robust and unbiased assessment of clinical outcomes and comorbidities in TKA patients with and without type 2 diabetes.

### Propensity score‐matched comparison of LOS and total hospital charges in TKA patients with and without type 2 diabetes

In the propensity score‐matched analysis shown in Table 3, there was no significant difference in the LOS between TKA patients with and without type 2 diabetes (mean 2.5 days in both groups, *p* = 0.86). However, total hospital charges remained significantly higher in the diabetic group, with a mean of $64,694 compared to $59,952 in the non‐diabetic group (*p* < 0.001).

**Table 3:** Comparison of hospitalization outcomes in propensity score‐matched cohorts of TKA patients with and without type 2 diabetes.

	Patients without type 2 diabetes	Patients with type 2 diabetes	Significance
Length of stay mean in days	2.5 (Std. deviation 1.6)	2.5 (Std. deviation 1.9)	*p* = 0.86
Total charges mean in $	59,952 (Std. deviation 35282)	64,694 (Std. deviation 40,918)	*p* < 0.001

Abbreviation: TKA, total knee arthroplasty.

### Elevated risk ratios (RRs) for general complications in TKA patients with type 2 diabetes compared to propensity score‐matched patients without type 2 diabetes

**Figure 2:**
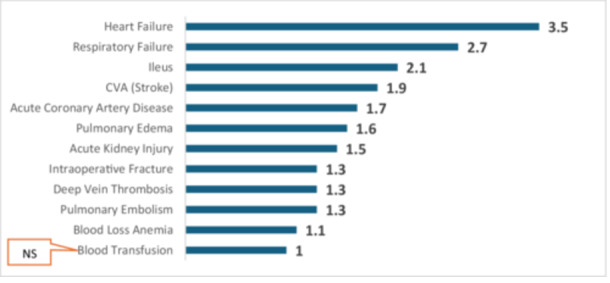
Risk ratios for general complications in TKA patients with type 2 diabetes compared to matched non‐diabetic patients. All complications shown are significant at *p* < 0.001 except blood transfusion (ns = not significant, *p* = 0.54). TKA, total knee arthroplasty.

Figure 2 illustrates the RRs for various general complications in TKA patients with type 2 diabetes compared to propensity score‐matched patients without diabetes. The results demonstrate significantly elevated risks for numerous complications (*p* < 0.001 in all except blood transfusion with *p* value of 0.54).

For blood loss anaemia, the RR is 1.1, with a 95% confidence interval (CI) of 1.1–1.1. Both pulmonary embolism and deep vein thrombosis show an increased risk, each with an RR of 1.3 (95% CI: 1.2–1.4). The RR for intraoperative fractures is also elevated at 1.3 (95% CI: 1.2–1.3). AKI shows a higher risk, with an RR of 1.5 (95% CI: 1.4–1.5), while pulmonary oedema demonstrates an increased risk with an RR of 1.6 (95% CI: 1.4–1.8). For acute coronary artery disease, the RR is 1.7 (95% CI: 1.5–1.9).

The risk for stroke (CVA) is notably elevated, with an RR of 1.9 (95% CI: 1.5–2.4). Ileus further shows an increased risk with an RR of 2.1 (95% CI: 1.9–2.4). The RR for respiratory failure is 2.7 (95% CI: 1.5–4.8), and heart failure demonstrates one of the highest observed risks with an RR of 3.5 (95% CI: 3.1–4.0). These elevated risks for complications persisted even after propensity matching, which included adjustments for hospital‐level variables such as hospital size, teaching status and geographic region, suggesting robustness of these findings independent of institutional characteristics.

### Elevated RRs for infection‐related complications and mortality in TKA patients with type 2 diabetes compared to propensity score‐matched patients without type 2 diabetes

**Figure 3:**
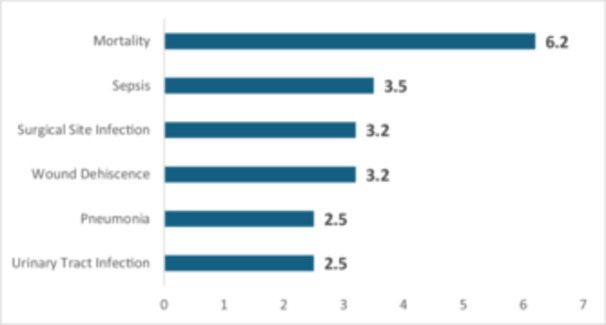
Risk ratios for infection‐related complications and mortality in TKA patients with type 2 diabetes compared to matched non‐diabetic patients. All differences shown are significant at *p* < 0.001. TKA, total knee arthroplasty.

Figure 3 presents the RR for infection‐related complications and mortality in TKA patients with type 2 diabetes compared to matched patients without diabetes. The results indicate a significantly elevated risk for infections and mortality among diabetic patients (*p* < 0.001 in all of the complications).

The RR for urinary tract infections is significantly elevated at 2.5 (95% CI: 2.3–2.6). Similarly, the RR for pneumonia is 2.5 (95% CI: 2.1–2.5). Wound dehiscence shows a higher risk, with an RR of 3.2 (95% CI: 2.5–4.1); surgical site infections have an identical RR of 3.2 (95% CI: 2.6–4.0).

For sepsis, the RR rises to 3.5 (95% CI: 2.9–4.1). Mortality demonstrates the highest observed risk, with an RR of 6.2 (95% CI: 4.2–9.1).

### Revision surgery outcomes in TKA patients with and without type 2 diabetes

The comparison of revision surgery outcomes between TKA patients with and without type 2 diabetes highlights significant differences as shown in Table 4. Patients with type 2 diabetes accounted for 24.8% of all revision surgeries.

**Table 4:** Revision surgery outcomes in TKA patients with and without type 2 diabetes.

Revision surgery	Patients without type 2 diabetes	Patients with type 2 diabetes	Significance
Total numbers	167,950 (75.2%)	55,290 (24.8%)	–
Age at revision (years)	65 (Std deviation 10.7)	66.4 (Std deviation 9.2)	*p* < 0.001
Total charges ($)	96,614 (Std deviation 90,612)	101,457 (Std deviation 88,358)	*p* = 0.015
Length of stay (days)	3.0 (Std deviation 2.8)	3.5 (Std deviation 3.1)	*p* < 0.001

Abbreviation: TKA, total knee arthroplasty.

The average age at revision was slightly higher in patients with diabetes (66.4 years, standard deviation 9.2) compared to those without diabetes (65 years, standard deviation 10.7; *p* < 0.001).

Total hospital charges were significantly higher in diabetic patients, with a mean of $101,457 compared to $96,614 in non‐diabetic patients (*p* = 0.015). LOS was also notably longer for patients with diabetes, averaging 3.5 days compared to 3.0 days in those without diabetes (*p* < 0.001).

### Aetiology for revision in TKA patients with and without type 2 diabetes

Table 5 compares the causes of revision in TKA patients with and without type 2 diabetes.

**Table 5:** Aetiology for revision in TKA patients with and without type 2 diabetes.

Aetiology for revision	Patients without type 2 diabetes	Patients with type 2 diabetes	Significance
Infection	21.0%	26.0%	*p* < 0.001
Mechanical loosening	23.2%	22.1%	
Pain	7.4%	7.1%	
Instability	12.5%	10.6%	
Wear of articular surface	2.1%	2.2%	
Periprosthetic fracture	0.9%	0.9%	
Arthrofibrosis	0.9%	1.0%	
Broken prosthesis	1.1%	0.9%	
Surgical wound complication	0.4%	0.6%	
Other mechanical complications	9.7%	8.8%	
Other/unspecified	20.8%	19.8%	

Abbreviation: TKA, total knee arthroplasty.

Infection was the leading cause of revision in both groups, with a significantly higher prevalence in patients with type 2 diabetes (26.0% vs. 21.0%; *p* < 0.001).

Other common causes, such as mechanical loosening and instability of the prosthesis, were slightly lower in diabetic patients (22.1% vs. 23.2% and 10.6% vs. 12.5%, respectively).

## DISCUSSION

### Key findings and clinical implications

The most important findings of this study were that patients with type 2 diabetes undergoing TKA experience significantly higher rates of post‐operative complications, including sepsis, heart failure, and surgical site infections, as well as increased total hospital charges and LOS compared to non‐diabetic patients [1, 3, 4, 6, 11, 13, 17, 22, 24, 26, 40]. Furthermore, diabetic patients undergoing revision TKA had a significantly higher risk of infection as the primary aetiology for revision, longer LOS and higher hospitalization costs [15, 21, 22, 26, 27, 31, 41]. These findings highlight the substantial impact of diabetes on both primary and revision TKA outcomes and reinforce the need for tailored perioperative management strategies for diabetic patients [6, 11, 13, 36].

These results align with recent meta‐analyses and large‐scale cohort studies, such as the systematic review by Hong et al., which demonstrated a significantly elevated risk of surgical complications in diabetic patients following TKA [11]. Furthermore, emerging evidence suggests that certain pharmacological interventions, such as glucagon‐like peptide‐1 receptor agonists (GLP‐1 RAs), may play a role in mitigating the progression of OA and delaying the need for TKA in diabetic patients [18].

Orthopaedic surgeons should consider individualized perioperative protocols for diabetic patients, including aggressive glycaemic optimization, cardiovascular screening, infection prevention measures and multidisciplinary collaboration to reduce the identified elevated risks.

### Increasing prevalence of type 2 diabetes in TKA patients

The growing prevalence of diabetes among TKA patients underscores the importance of optimizing perioperative care in this population. Our study found a steady increase in the proportion of diabetic patients undergoing TKA from 2016 to 2019, mirroring global trends in diabetes prevalence [28]. Previous studies have estimated that by 2030, nearly 50% of TKA patients will have diabetes [14, 28]. Given the well‐established link between diabetes and poorer surgical outcomes [10, 11, 17, 34], proactive management strategies are essential to improve perioperative safety and long‐term outcomes.

### Increased post‐operative complications and healthcare costs

Our study confirms that diabetic patients face significantly higher risks for post‐operative complications, including sepsis, pneumonia, respiratory failure, and surgical site infections [6, 11, 20, 22, 24, 40]. These findings are consistent with those of Rahbari et al., who reported an increased rate of wound dehiscence and infection in diabetic patients undergoing TKA [30]. Additionally, we found that diabetic patients incurred significantly higher hospital charges ($64,694 vs. $59,952, *p* < 0.001), further supporting the economic burden of diabetes in orthopaedic surgery [2, 3, 5, 11, 13, 22, 26, 29].

Our findings align with results from smaller‐scale prospective studies, further strengthening the evidence that diabetic patients undergoing TKA are at significantly increased risk of post‐operative complications. However, our nationwide analysis provides substantially stronger statistical power and generalizability, reinforcing the clinical relevance of our findings [2, 3, 13, 17].

### Increased risk of heart failure in diabetic patients undergoing TKA

One of the most concerning findings of this study is the markedly elevated risk of heart failure in diabetic patients undergoing TKA (RR = 3.5). The high prevalence of cardiovascular disease in diabetic populations likely contributes to this increased risk [16, 28]. Additionally, certain antidiabetic medications, such as dipeptidyl peptidase‐4 inhibitors, have been linked to increased hospitalization rates for heart failure [7, 16]. However, newer glucose‐lowering agents, such as sodium‐glucose cotransporter‐2 (SGLT2) inhibitors and GLP‐1 RAs, have shown potential benefits in reducing cardiovascular complications and may be valuable in preoperative risk mitigation [5, 6, 18]. Further studies are needed to assess the impact of optimizing preoperative diabetes management on reducing cardiovascular risks in TKA patients [6, 12, 16].

### Revision surgery and long‐term outcomes

Diabetic patients undergoing revision TKA had significantly worse outcomes, with higher infection rates (26.0% vs. 21.0%) and increased hospital costs ($101,457 vs. $96,614) [21, 22, 26]. The higher infection burden in diabetic patients aligns with findings from Palmer et al., who questioned the utility of HbA1c alone as a predictor of periprosthetic joint infection and suggested a need for additional perioperative glycaemic control strategies [26]. Given the increased risk of infection‐related revision in diabetic patients, implementing strict perioperative infection prevention protocols, including glycaemic optimization, may help mitigate these risks [8, 14, 30, 35].

### Preoperative strategies to optimize outcomes in diabetic patients undergoing TKA

Based on our findings and the current literature, the following preoperative interventions may help reduce the risks associated with diabetes in TKA patients:

1. Preoperative glycaemic optimization:

- HbA1c should be optimized to <7.5% before surgery, as higher levels have been linked to increased complications [8, 12, 37].
- Short‐term intensive insulin therapy may be beneficial for poorly controlled diabetic patients prior to TKA [12, 26, 30].

2. Cardiovascular risk assessment:

- Routine cardiac evaluation, including echocardiography and stress testing, should be considered for diabetic patients at high risk of heart failure [7, 16].
- Consideration of cardioprotective agents such as SGLT2 inhibitors for high‐risk patients [5, 6].

3. Infection prevention measures:

- Routine screening and decolonization for *Staphylococcus aureus* in diabetic patients preoperatively [14, 30].
- Optimization of perioperative antibiotic prophylaxis based on risk stratification [8, 35].

4. Nutritional and weight optimization:

- Weight loss interventions, including preoperative dietary counselling or bariatric surgery for morbidly obese diabetic patients, have been shown to improve post‐operative outcomes [9, 18, 32].

5. Multidisciplinary prehabilitation programs:

- Collaboration between orthopaedic surgeons, endocrinologists, and anesthesiologists for tailored perioperative management [20, 22, 29].

Additionally, emerging evidence supports the use of novel antidiabetic agents such as GLP‐1 RAs and SGLT2 inhibitors, which may reduce cardiovascular and infection‐related complications due to their glucose‐independent cardioprotective and anti‐inflammatory effects. Implementation of multidisciplinary prehabilitation programs involving endocrinologists, anesthesiologists and surgeons may also significantly reduce perioperative risk by optimizing diabetes control, cardiovascular status and nutritional health preoperatively.

## LIMITATIONS

Our study has several limitations. Administrative databases such as NIS inherently carry the limitation of potential inaccuracies due to miscoding, underreporting or incomplete documentation, potentially affecting the accuracy of recorded diagnoses and complications. While the large sample size and standardized methodology minimize these concerns, the possibility of misclassification bias must be considered when interpreting our findings [21]. Additionally, the NIS only provides data for the in‐hospital period, limiting the ability to assess long‐term outcomes [11, 22]. Future research should involve long‐term follow‐up to gain a deeper understanding of the full impact of diabetic history on TKA surgery outcomes [6, 24, 38, 43].

Another limitation is the differences between haemoglobin A1c levels of diabetic patients that have not been considered. Different haemoglobin A1c levels of diabetic patients have diverse effects on post‐operative glucose levels [26, 33, 37]. As we mentioned before, there is a correlation between post‐operative glucose levels and post‐operative outcomes [24, 38]. Those might affect the outcomes if we could include it in our research.

Despite the limitations of this study, its advantages include the use of a large, nationally representative data set and the application of robust statistical methods to ensure the reliability and validity of the results [21]. The findings contribute to the growing body of evidence supporting the implications of diabetic history before TKA in improving patient outcomes and reducing healthcare costs [10, 13, 20, 29].

## FUTURE STUDIES

Future studies should explicitly differentiate diabetes subtypes, particularly insulin‐dependent versus non‐insulin‐dependent diabetes, as these subgroups may have different risk profiles and outcomes. Additionally, prospective research incorporating detailed perioperative glycaemic control data (such as continuous glucose monitoring and HbA1c levels) would provide valuable insights, enabling more targeted perioperative management guidelines to reduce complication rates and healthcare costs for diabetic patients undergoing TKA.

## CONCLUSION

In conclusion, our nationwide study demonstrates diabetic patients undergoing TKA face significantly greater post‐operative complication risks, healthcare costs and revision surgery burden. Orthopaedic teams should implement actionable strategies such as personalized glycaemic optimization protocols, cardiovascular screening, rigorous infection prevention measures and multidisciplinary perioperative care to effectively mitigate these risks and improve patient outcomes. Further prospective research is needed to optimize long‐term diabetic management strategies surrounding TKA procedures.

## AUTHOR CONTRIBUTIONS

Yaara Berkovich and Ela Cohen Nissan contributed to the majority of the manuscript writing and literature review. David Maman performed the statistical analysis, data interpretation and contributed to manuscript revision. Michael Tobias Hirschmann assisted in writing the manuscript and provided senior mentorship throughout the project. Yaniv Yonai and Yaniv Steinfeld contributed to the clinical interpretation and manuscript editing. Yaron Berkovich supervised the entire project and served as the principal investigator for all stages of the research. All authors reviewed and approved the final version of the manuscript.

## CONFLICT OF INTEREST STATEMENT

The authors declare no conflicts of interest.

## ETHICS STATEMENT

The study was conducted under an exempt status granted by the Institutional Review Board (IRB). Due to the use of a de‐identified data set from the National Inpatient Sample (NIS), the requirement for informed consent was waived. The research adhered to the ethical principles outlined in the Declaration of Helsinki. Consent was not required for this study, as it utilized a de‐identified data set, with no direct human participation involved that would necessitate consent.

## Data Availability

The data set analyzed during the current study is available for purchase from the Healthcare Cost and Utilization Project (HCUP) National Inpatient Sample (NIS), sponsored by the Agency for Healthcare Research and Quality (AHRQ), at www.hcup-us.ahrq.gov.

## References

[1] Baker PN , Rushton S , Jameson SS , Reed M , Gregg P , Deehan DJ . Patient satisfaction with total knee replacement cannot be predicted from pre‐operative variables alone: a cohort study from the National Joint Registry for England and Wales. Bone Joint J. 2013;95(10):1359–1365. 24078532 10.1302/0301-620X.95B10.32281

[2] Carender CN , Fruth KM , Lewallen DG , Berry DJ , Abdel MP , Bedard NA . Obesity and primary total knee arthroplasty: the absolute versus relative risk of periprosthetic joint infection at 15 years. J Arthroplasty. 2025;40(5):1204.e4–1209.e4. 39442895 10.1016/j.arth.2024.10.064

[3] Chandrupatla S , Rumalla K , Singh JA . Association between diabetes mellitus and total hip arthroplasty outcomes: an observational study using the US National Inpatient Sample. BMJ Open. 2024;14(7):085400. 10.1136/bmjopen-2024-085400 PMC11404163 39038867

[4] Choi YJ , Ra HJ . Patient satisfaction after total knee arthroplasty. Knee Surg Relat Res. 2016;28(1):1–15. 26955608 10.5792/ksrr.2016.28.1.1 PMC4779800

[5] Chong K , Chang JK , Chuang LM . Recent advances in the treatment of type 2 diabetes mellitus using new drug therapies. Kaohsiung J Med Sci. 2024;40(3):212–220. 38183334 10.1002/kjm2.12800 PMC11895656

[6] Eitner A , Culvenor AG , Wirth W , Schaible HG , Eckstein F . Impact of diabetes mellitus on knee osteoarthritis pain and physical and mental status: data from the osteoarthritis initiative. Arthritis Care Res. 2021;73(4):540–548. 10.1002/acr.24173 32105401

[7] Fadini GP , Avogaro A , Degli Esposti L , Russo P , Saragoni S , Buda S , et al. Risk of hospitalization for heart failure in patients with type 2 diabetes newly treated with DPP‐4 inhibitors or other oral glucose‐lowering medications: a retrospective registry study on 127,555 patients from the Nationwide OsMed Health‐DB Database. Eur Heart J. 2015;36(35):2454–2462. 26112890 10.1093/eurheartj/ehv301

[8] Godshaw BM , Ojard CA , Adams TM , Chimento GF , Mohammed A , Waddell BS . Preoperative glycemic control predicts perioperative serum glucose levels in patients undergoing total joint arthroplasty. J Arthroplasty. 2018;33(7 Suppl):76–80. 10.1016/j.arth.2018.02.071 29576485

[9] Han HS , Kang SB . Relations between long‐term glycemic control and postoperative wound and infectious complications after total knee arthroplasty in type 2 diabetics. Clin Orthop Surg. 2013;5(2):118–123. 23730475 10.4055/cios.2013.5.2.118 PMC3664670

[10] Helbing J , Farley B , Gu A , Zhao AY , Siram G , Stein B , et al. Diabetes mellitus and total ankle arthroplasty complications. Foot Ankle Int. 2024;45(4):320–327. 38327200 10.1177/10711007241226929

[11] Hong SH , Kwon SC , Lee JH , Moon S , Kim JI . Influence of diabetes mellitus on postoperative complications after total knee arthroplasty: a systematic review and meta‐analysis. Medicina (Kaunas). 2024;60(11):1757. 39596942 10.3390/medicina60111757 PMC11595993

[12] Kavin M , Yayac M , Grosso MJ , Courtney PM . Preoperative hemoglobin A1c >7.5 is associated with increased bundled payment costs in total hip and knee arthroplasties. J Am Acad Orthop Surg. 2021;29(22):970–976. 33306559 10.5435/JAAOS-D-20-00944

[13] Kheir MM , Tan TL , Kheir M , Maltenfort MG , Chen AF . Postoperative blood glucose levels predict infection after total joint arthroplasty. J Bone Joint Surg Am. 2018;100(16):1423–1431. 30106824 10.2106/JBJS.17.01316

[14] Lai J , Li Q , He Y , Zou S , Bai X , Rastogi S . Glycemic control regimens in the prevention of surgical site infections: a meta‐analysis of randomized clinical trials. Front Surg. 2022;9:855409. 35402490 10.3389/fsurg.2022.855409 PMC8990940

[15] Laver L , Maman D , Hirschmann MT , Mahamid A , Bar O , Steinfeld Y , et al. Big data analysis reveals significant increases in complications, costs, and hospital stay in revision total knee arthroplasty compared to primary TKA. Knee Surg Sports Traumatol Arthrosc. 2025;33(3):1015–1024. 39382040 10.1002/ksa.12499 PMC11848982

[16] Lehrke M , Marx N . Diabetes mellitus and heart failure. Am J Cardiol. 2017;120(1 Suppl):37. 10.1016/j.amjcard.2017.05.014 28606342

[17] Li S , Si H , Zhang S , Xu J , Liu Y , Shen B . Does diabetes mellitus impair the clinical results of total knee arthroplasty under enhanced recovery after surgery? J Orthop Surg. 2023;18(1):490. 10.1186/s13018-023-03982-4 PMC10332043 37430329

[18] Lin CP , Chung CH , Lu CH , Su SC , Kuo FC , Liu JS , et al. Glucagon‐like peptide‐1 receptor agonists therapy to attenuate the risk of knee osteoarthritis and total knee replacement in type 2 diabetes mellitus: a nation‐wide population‐based cohort study. Medicine. 2025;104(6):41243. 10.1097/MD.0000000000041243 PMC11813052 39928811

[19] Liu SS , Della Valle AG , Besculides MC , Gaber LK , Memtsoudis SG . Trends in mortality, complications, and demographics for primary hip arthroplasty in the United States. Int Orthop. 2009;33:643–651. 18461326 10.1007/s00264-008-0549-4 PMC2903109

[20] Luong LM , Kostyun RO , Witmer DK , Grady‐Benson JC . The influence of multiple modifiable risk factors on 30‐day readmissions and 90‐day major complications after a total hip and knee arthroplasty: an analysis of a large claims database. J Am Acad Orthop Surg Glob Res Rev. 2025;9(2):e24.00151. 10.5435/JAAOSGlobal-D-24-00151 PMC11781773 39899738

[21] Maman D , Liba G , Hirschmann MT , Ben Zvi L , Fournier L , Steinfeld Y , et al. Predictive analysis of economic and clinical outcomes in total knee arthroplasty: identifying high‐risk patients for increased costs and length of stay. Knee Surg Sports Traumatol Arthrosc. 2025;33(5):1754–1762. 39629972 10.1002/ksa.12547 PMC12022826

[22] Houdek MT , Wyles CC , Labott JR , Rose PS , Taunton MJ , Sim FH . Durability of hemiarthroplasty for pathologic proximal femur fractures. J Arthroplasty. 2017;32:3607–3610. 28735800 10.1016/j.arth.2017.06.040

[23] Memtsoudis SG , Della Valle AG , Besculides MC , Gaber L , Laskin R . Trends in demographics, comorbidity profiles, in‐hospital complications and mortality associated with primary knee arthroplasty. J Arthroplasty. 2009;24(4):518–527. 18534410 10.1016/j.arth.2008.01.307

[24] Na A , Jansky L , Gugala Z . Clinical characteristics of patients with type 2 diabetes mellitus receiving a primary total knee or hip arthroplasty. J Diabetes Res. 2019;2019:9459206. 31828171 10.1155/2019/9459206 PMC6885807

[25] Nieves‐Plaza M , Castro‐Santana LE , Font YM , Mayor AM , Vilá LM . Association of hand or knee osteoarthritis with diabetes mellitus in a population of Hispanics from Puerto Rico. J Clin Rheumatol. 2013;19:1–6. 23319016 10.1097/RHU.0b013e31827cd578 PMC3815459

[26] Palmer RC , Telang SS , Ball JR , Wier J , Lieberman JR , Heckmann ND . The limited utility of hemoglobin A1c as a predictor for periprosthetic joint infection following total joint arthroplasty: a continuous variable analysis. J Arthroplasty. 2025;S0883–5403(5):00019–1. 10.1016/j.arth.2025.01.004 39814114

[27] Patti AM , Rizvi AA , Giglio RV , Stoian AP , Ligi D , Mannello F . Impact of glucose‐lowering medications on cardiovascular and metabolic risk in type 2 diabetes. J Clin Med. 2020;9(4):912. 32225082 10.3390/jcm9040912 PMC7230245

[28] Pop‐Busui R , Januzzi JL , Bruemmer D , Butalia S , Green JB , Horton WB , et al. Heart failure: an underappreciated complication of diabetes. A consensus report of the American Diabetes Association. Diabetes Care. 2022;45(7):1670–1690. 35796765 10.2337/dci22-0014 PMC9726978

[29] Power JD , Perruccio AV , Canizares M , Davey JR , Gandhi R , Mahomed NN , et al. The impact of diabetes status on pain and physical function following total joint arthroplasty for hip and knee osteoarthritis: variation by sex and body mass index. Sci Rep. 2024;14(1):11152. 38750058 10.1038/s41598-024-61847-0 PMC11096302

[30] Rahbari H , Ahmadi M , Doreh MA , Mahmoudi S , Ghaemmaghami P , Fereidouni A . Comparison of surgical wound infection and dehiscence following the use of two methods of nylon sutures and skin staples in staples in diabetic mellitus patients undergoing total knee arthroplasty surgery: a randomized clinical trial study. BMC Musculoskelet Disord. 2025;26(1):70. 39828687 10.1186/s12891-024-08263-7 PMC11744891

[31] Robertson F , Geddes J , Ridley D , McLeod G , Cheng K . Patients with Type 2 diabetes mellitus have a worse functional outcome post knee arthroplasty: a matched cohort study. Knee. 2012;19(4):286–289. 21715174 10.1016/j.knee.2011.06.001

[32] Roglic G . WHO Global report on diabetes: a summary. Int J Noncommun Dis. 2016;1(1):3–8.

[33] Saudek CD , Derr RL , Kalyani RR . Assessing glycemia in diabetes using self‐monitoring blood glucose and hemoglobin A1c. JAMA. 2006;295(14):1688–1697. 16609091 10.1001/jama.295.14.1688

[34] Schett G , Kleyer A , Perricone C , Sahinbegovic E , Iagnocco A , et al. Diabetes is an independent predictor for severe osteoarthritis: results from a longitudinal cohort study. Diabetes Care. 2013;36:403–409. 23002084 10.2337/dc12-0924 PMC3554306

[35] Shen D , Sun S , Mu Z , Yuefu D . The maximum threshold value for HbA1c in diabetic patients undergoing elective total knee arthroplasty. J Coll Physicians Surg Pak. 2024;34(9):1073–1078. 39262008 10.29271/jcpsp.2024.09.1073

[36] Shichman I , Oakley CT , Konopka JA , Rozell JC , Schwarzkopf R , Lajam CM . Preoperatively elevated HbA1c levels can meaningfully improve following total joint arthroplasty. Arch Orthop Trauma Surg. 2023;143(8):5425–5435. 36703084 10.1007/s00402-023-04765-6

[37] Tarabichi M , Shohat N , Kheir MM , Adelani M , Brigati D , Kearns SM , et al. Determining the threshold for HbA1c as a predictor for adverse outcomes after total joint arthroplasty. J Arthroplasty. 2017;32(9 Suppl):263–267.e1. 28662955 10.1016/j.arth.2017.04.065

[38] Vervullens S , Meert L , Smeets RJEM , Verbrugghe J , Baert I , Rahusen FTG , et al. Preoperative glycaemic control, number of pain locations, structural knee damage, self‐reported central sensitisation, satisfaction and personal control are predictive of 1‐year postoperative pain, and change in pain from pre‐ to 1‐year post‐total knee arthroplasty. Knee Surg Sports Traumatol Arthrosc. 2025;33(1):201–219. 38751081 10.1002/ksa.12265 PMC11716348

[39] Wang HJ , Giambini H , Chen JW , Wang QS , Hou HG , Luo SM , et al. Diabetes mellitus accelerates the progression of osteoarthritis in streptozotocin‐induced diabetic mice by deteriorating bone microarchitecture, bone mineral composition, and bone strength of subchondral bone. Ann Transl Med. 2021;9(9):768. 34268381 10.21037/atm-20-6797 PMC8246216

[40] Webb ML , Golinvaux NS , Ibe IK , Bovonratwet P , Ellman MS , Grauer JN . Comparison of perioperative adverse event rates after total knee arthroplasty in patients with diabetes: insulin dependence makes a difference. J Arthroplasty. 2017;32:2947–2951. 28559194 10.1016/j.arth.2017.04.032

[41] Wright AK , Carr MJ , Kontopantelis E , Leelarathna L , Thabit H , Emsley R , et al. Primary prevention of cardiovascular and heart failure events with SGLT2 inhibitors, GLP‐1 receptor agonists, and their combination in type 2 diabetes. Diabetes Care. 2022;45(4):909–918. 35100355 10.2337/dc21-1113

[42] Yang G , Meng F , Liu Y , Kong L , Shen Y . Diabetes mellitus and risk of deep vein thrombosis after total knee replacement: a meta‐analysis of cohort studies. Int J Clin Exp Med. 2015;8:9086–9092. 26309562 PMC4537981

[43] Zhuang T , Shapiro LM , Fogel N , Richard MJ , Gardner MJ , Kamal RN . Perioperative laboratory markers as risk factors for surgical site infection after elective hand surgery. J Hand Surg. 2021;46(8):675–684. 10.1016/j.jhsa.2021.04.001 34016493

